# Higher ETV5 Expression Associates With Poor 5-Florouracil-Based Adjuvant Therapy Response in Colon Cancer

**DOI:** 10.3389/fphar.2020.620811

**Published:** 2021-02-09

**Authors:** Anil K. Giri

**Affiliations:** Institute for Molecular Medicine Finland (FIMM), University of Helsinki, Helsinki, Finland

**Keywords:** ETV5, ETV4, 5-fluorouracil, colon cancer, PEA3

## Abstract

Discovery of markers predictive for 5-Fluorouracil (5-FU)-based adjuvant chemotherapy (adjCTX) response in patients with locally advanced stage II and III colon cancer (CC) is necessary for precise identification of potential therapy responders. PEA3 subfamily of ETS transcription factors (ETV1, ETV4, and ETV5) are upregulated in multiple cancers including colon cancers. However, the underlying epigenetic mechanism regulating their overexpression as well as their role in predicting therapy response in colon cancer are largely unexplored. In this study, using gene expression and methylation data from The Cancer Genome Atlas (TCGA) project, we showed that promoter DNA methylation negatively correlates with ETV4 expression (*ρ* = −0.17, *p* = 5.6 × 10^–3^) and positively correlates with ETV5 expression (*ρ* = 0.22, *p* = 1.43 × 10^–4^) in colon cancer tissue. Further, our analysis in 1,482 colon cancer patients from five different cohorts revealed that higher ETV5 expression associates with shorter relapse-free survival (RFS) of adjCTX treated colon cancer patients (Hazard ratio = 2.09–5.43, *p* = 0.004–0.01). The present study suggests ETV5 expression as a strong predictive biomarker for 5-FU-based adjCTX response in stage II/III CC patients.

## Introduction

Colon cancer (CC) is the fourth most commonly diagnosed cancer (2 million cases in 2018) globally and kills nearly 1 million people annually (Arnold et al., 2017) mostly due to the spread of tumor cells to other secondary organs (e.g., liver) in the later stage (stage IV) of the disease (Yu et al., 2019). Therefore, successful treatment of the early-stage (stage I, II, and III) cancer is necessary in order to prevent disease progression and improve the overall survival of the patients (Argiles et al., 2020). Usually, the early-stage patients are cured by surgical removal of the tumor only without the use of chemotherapy, however, systematic use of 5-fluorouracil (5-FU)-based-adjCTX is recommended for stage II cases with high risk (e.g., with perineural invasion and poor histological differentiation) of reoccurrence and stage III patients (Casadaban et al., 2016; Argiles et al., 2020). The use of adjCTX cures only 20% of additional stage III patients over surgery alone (cures 50% of cases) and improves the chance of 10-year overall survival only by 10–20% in stage II patients (Casadaban et al., 2016)^.^ Further, it incurs considerable toxicity (e.g., myelosuppression, diarrhea) and economic cost to the patients (Breugom et al., 2015; Auclin et al., 2017). The higher toxicity and low efficacy of 5-FU-based adjCTX demand novel and reliable molecular markers that can predict the treatment response in early-stage (II and III) patients and help to stratify patients with different responses (Giri and Aittokallio, 2019; Giri et al., 2019).

Attempts to predict response for adjuvant chemotherapy have identified molecular alterations (e.g., microsatellites status (Ribic et al., 2003), TP53 mutations (Kandioler et al., 2015), genetic polymorphism in MTHFR (Nahid et al., 2018) and DPYD (Henricks et al., 2017; Hariprakash et al., 2018) as a predictive marker for 5-FU-based adjCTX response. Further, recent studies exploring gene expression signatures as predictive markers for treatment response in colon cancer have identified ESR1 (Ye et al., 2020), and CD8 (Allen et al., 2018) expression as predictors for 5-FU-based adjCTX response in CRC. However, none of the identified markers can successfully segregate the responders from nonresponders suggesting a need for additional novel markers predictive of adjCTX response (Oh and Joo, 2020).

ETV1, ETV4, and ETV5 are the members of the polyoma enhancer activator 3 (PEA3) subfamily of E26 transformation-specific (ETS) domain-containing transcription factors. They promote cancer cell proliferation and survival in solid tumors including gastric (Keld et al., 2011), ovarian (Llaurado et al., 2012), and colon (Cheng et al., 2019) cancers and are being targeted for therapy (Hsing et al., 2020). However, the role of epigenetic mechanisms especially DNA methylation in regulating their expression in colon cancer (Llaurado et al., 2012) is largely unexplored. Further, the available evidence suggests that PEA3 subfamily members could be a potential biomarker for therapy response against CC (Llaurado et al., 2012), and their role in predicting adjCTX response has not been explored in any cancers including CC. The current study explores the role of DNA methylation in regulating PEA3 members gene expression using The Cancer Genome Atlas (TCGA) colon adenocarcinoma cohort. Further, we explored the PEA3 subfamily ETS transcription factors as predictive biomarkers for adjCTX response in CC patients. Our analysis identified and validated ETV5 expression as a predictive marker for 5-FU-based adjCTX response in stage II and II colon cancer patients.

## Materials and Methods

### Processing of Clinical and Expression Data From Publicly Available Cohorts

#### The Cancer Genome Atlas (TCGA) Cohort

The expectation-maximization genes normalized RNA-Seq data for 328 colon adenocarcinomas patients’ samples (41 normal and 287 cancerous tissue) profiled in the TCGA project were downloaded using the Firehose tool (http://gdac.broadinstitute.org/). The data was further normalized using voom function in the limma package (Ritchie et al., 2015), and Z-transformed before the differential and correlation analyses. We also downloaded methylation data for 482,481 CpGs generated using Infinium HumanMethylation450 Beadchip for 38 normal and 297 cancerous tissue samples. Methylation status at a CpG site was measured as beta value (*β*), which is the ratio of the methylated probe intensity and the overall intensity (sum of methylated and unmethylated probe intensities designed for a particular CpG in 450K beadchip). *β* ranges from 0 to 1, indicating no methylation (*β* = 0) to complete methylation of the CpGs (*β* = 1). In all the analyses, we performed appropriate quality control of the published data before their downstream analysis as described previously (Giri et al., 2017; Giri and Aittokallio, 2019). Briefly, we removed all the CpGs with missing values and CpGs assessed by probes that have a tendency of cross-hybridization, as specified in the supplementary file of Chen et al., 2013. We used BMIQ normalization (Teschendorff et al., 2013) to remove any possible bias due to design differences in the type of probes (the type I and type II probes) present in the Illumina 450K platform before averaging of probes in the promoter region. The average methylation of probes between 1,500 bases upstream of the transcription start site (TSS) was defined as promoter methylation level for a gene.

#### The French National Cartes d’Identité des Tumeurs Program Cohort

We downloaded the clinical and normalized gene expression profile of 472 stage II and III colon cancer patients out of 585 samples collected under The Cartes d’Identité des Tumeurs (CIT) program from the Gene Expression Omnibus (GEO) platform (GSE39582, Marisa et al., 2013). These patients with primary tumors have been treated with 5-Fluorouracil based adjuvant chemotherapy after surgery and monitored for relapse (distant and/or locoregional recurrence; median follow-up of 51.5 months) at the Institut Gustave Roussy (Villejuif), the Hospital Saint Antoine (Paris), the Hospital Europe’en Georges Pompidou (Paris), the Hospital de Hautepierre (Strasbourg), the Hospital Purpan (Toulouse), and the Institut Paoli-Calmettes (Marseille), and the Center Antoine Lacassagne (Nice) between 1987 and 2007. Clinical and pathological data were extracted from the medical records and centrally reviewed for the purpose of this study. The recurrence-free survival (RFS) for the patients has been calculated as the time from surgery to the first recurrence. Patients have been staged according to the American Joint Committee on cancer tumor node metastasis (TNM) staging system (American Joint Committee on Cancer, 1997). The locations of the tumor have been noted as distal and proximal based on their anatomical positions. The gene expression data have been generated on Affymetrix U133 Plus 2.0 chips and normalized using the robust multi-array average method implemented in the R package affy. Gene expression was summarized as the average expression levels of all the probes of the genes and was used for differential and survival analysis.

#### H. Lee Moffitt Cancer Center Cohort

We also downloaded gene expression and clinical data for 177 colon cancer patient’s data (GSE17536) treated with adjuvant therapy at the H. Lee Moffitt Cancer Center (MCC; Tampa, FL). Out of 177 patients, we used 56 stage III patients’ data for downstream analysis (Smith et al., 2010). These patients have been treated with adjuvant therapy and disease-free survival (DFS), as well as disease-specific survival (DSS), have been reported as clinical endpoints. In order to generate the gene expression data, representative sections of fresh tissue specimens were flash-frozen in liquid nitrogen and stored at −80°C until RNA isolation. RNA was purified with the use of the RNeasy kit (QIAGEN, Valencia, CA). Human RNA samples were hybridized to Affymetrix arrays (Human Genome U133 Plus 2.0 GeneChip Expression Arrays).

#### Metastasis Cohort (GSE72970)

Furthermore, we downloaded expression data (GSE72970) for tumor samples from 143 patients collected by Del Rio et al., 2017. We selected 63 colon cancer patients treated with 5-FU based adjCTX and 21 untreated patients for our analysis. The patients had metastatic colon cancer and did not receive any chemotherapy treatment before primary tumor resection. Tumor response was evaluated according to RECIST 1.0 recommendations for the assessment of cancer treatment in solid tumors as described previously. Overall survival (OS) and progression-free survival (PFS) have been reported as treatment responses. PFS was defined as the time from the beginning of first-line metastatic treatment until recurrence or death. Alive patients without progression were censored at the date of the last contact. OS was calculated from the beginning of first-line treatment until death. Gene expression data have been generated using human genome U133 Plus 2.0 arrays (Affymetrix Inc., Santa Clara, CA, United States). The details of the study participants have been sown in Supplementary Table S1.

#### GSE14333 Cohort

Additionally, we also downloaded the clinical and normalized gene expression data (GSE14333) for 290 colorectal patients published by Jorissen et al. (2009). The gene expression data have been collected from specimens derived from primary carcinomas tissue-sections snap-frozen in liquid nitrogen immediately after surgery. RNA has been isolated and hybridized on human genome U133 Plus 2.0 arrays. The patients have received standard adjCTX (either single-agent 5-fluorouracil/capecitabine or 5-fluorouracil and oxaliplatin, Jorissen et al., 2009). Disease-free survival (DFS) has been calculated as the duration from surgical operation to cancer recurrence, second cancer, or death from any cause. The grading for tumor stages has been determined using AJCC cancer staging manual and the position of the tumor has been noted as left, right, colon, and rectum (American Joint Committee on Cancer, 1997). We removed the rectal cancer patients and also cases who received postoperative chemoradiotherapy (50.4 Gy in 28 fractions) concurrent with 5-fluorouracil from our analysis. Finally, we analyzed the effect of ETV5 expression over DFS in 61 adjCTX treated patients with Duke stage B or C.

### Survival Analysis

The effect of ETV5 expression over RFS or DFS was determined using the Cox regression analysis. The hazard ratio has been calculated as the exponential of the regression coefficient obtained from the fitted regression model. The significance of the model was tested using the log-rank test.

### Enrichment Analysis

The biological pathway enrichment for 22 genes against the human genome as the background was performed using Genecodis 4. 0 (Tabas-Madrid et al., 2012) and False Discovery Rate adjusted hypergeometric *p*-values were used to identified enriched pathways.

### Statistical Analysis

All the statistical analysis has been performed using R version 3.5.3. The Non-parametric Wilcoxon rank test has been used to compare the expression profiles between two groups. The survival analysis has been performed using Cox-proportional regression as implemented in the “survival” package and the survival plots have been drawn using “ggplot” and “GGally” package in R.

Calculation of oncotype DX recurrence score (RS): In order to compare the prediction ability of ETV5, we correlated the ETV5 expression in the treated patients with the oncotype DX recurrent score (RS) across different datasets. Oncotype DX recurrent score (RS) was calculated using the normalized gene expression of seven genes from three gene groups as described below (Clark-Langone et al., 2010)

1. cell proliferation group-MK167, MYBL2, and MYC,
2. stroma activation group-BGN, INHBA, and FAP, and
3. genotoxic stress pathway-GADD45B.

The unscaled recurrence score (RS) was calculated as

RS = 0.1263 × Stromal Group Score − 0.3158 × Cell Cycle Group Score + 0.3406 × GADD45B

where

Stromal Group Score = (BGN+FAP+INHBA)/3 and Cell Cycle Group Score = (MYBL2+Ki-67+MYC)/3

The unscaled RS (recurrence score) were then rescaled be between 0 and 100 as given below

The RS score is

0 if 44.16* (RS+ 0.30) < 0 OR

44.16 *(RS +0.30) if 0> 44.16 (RS +0.30) <100 OR

100 if 44.16 (RS +0.30) > 100

## Results

### Cancer Tissue Has Higher Expression of ETV4 and ETV5 Genes That Correlate With Promoter Methylation in Colon Cancer Patients

**FIGURE 1:**
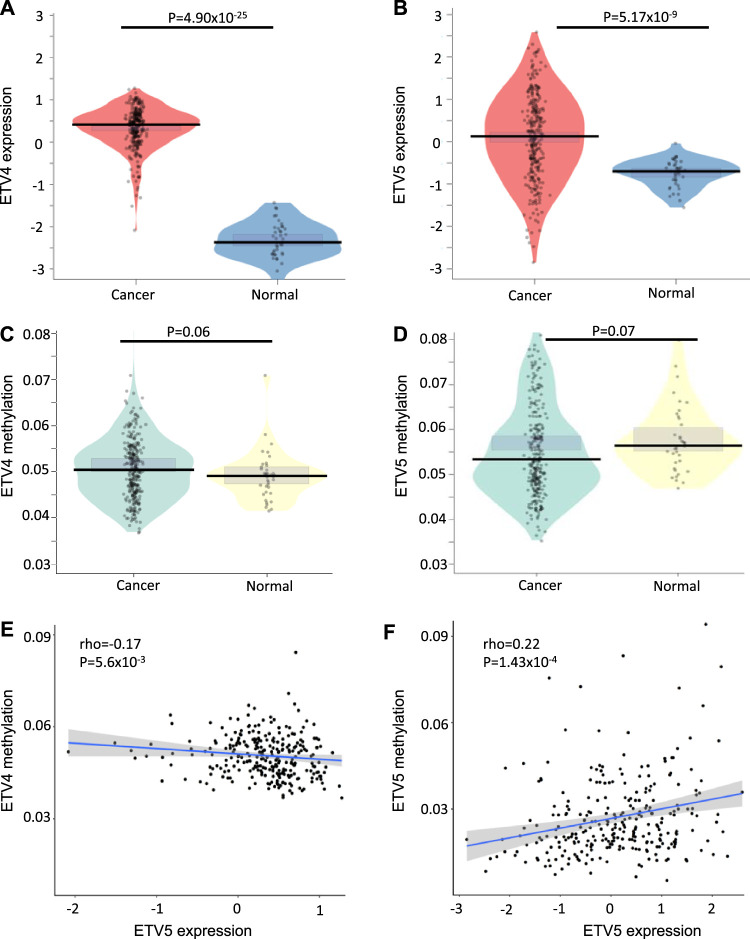
DNA methylation regulates ETV4 and ETV5 expression in colon cancer. ETV4 **(A)** and ETV5 **(B)** are overexpressed in cancerous tissue compared to healthy tissue in TCGA samples. Violin plot showing promoter methylation difference between cancer and normal tissue for ETV4 **(C)** and ETV5 **(D)** genes. The *p*-value has been calculated using the Wilcoxon nonparametric test. Scatterplot showing spearman’s correlation between promoter methylation and expression levels of ETV4 **(E)** and ETV5 **(F)** in the cancerous tissue of the CC patients from TCGA data. The Spearman correlation coefficient and respective *p*-values have been shown in the figure.

First, we compared the expression level of ETV1, ETV4, and ETV5 between the normal and cancerous tissue in TCGA data and observed higher expression of only ETV4 (Wilcox test *p* = 4.90 × 10^–25^) and ETV5 (Wilcox test *p* = 5.17 × 10^–9^) in tumor suggesting their possible role in tumor biology (Figures 1A,B) Further, our analysis revealed that promoter methylation negatively correlates with ETV4 expression (*ρ* = -0.17, *p* = 5.6 × 10^–3^) whereas positively correlates with ETV5 expression (*ρ* = 0.22, p = 1.43 × 10^–4^) in cancer tissue suggesting that DNA methylation play a strong role in regulating ETV5 and ETV4 expression in colon cancer tissue (Figures 1C‐F).

#### ETV5 Correlates With Disease Free Survival Of Stage II/III Patients Treated With Adjuvant Chemotherapy.

After observing that ETV4 and ETV5 are overexpressed in colon cancerous tissue from TCGA cohort, we studied their role in predicting adjCTX response in colon cancer using three publicly available datasets. We used cox-regression with relapse/progression-free survival as treatment outcomes and observed that higher ETV5 expression is a strong and selective predictor for poor RFS (HR= 2.29, *p* = 0.00178) in treated patients in the CIT program cohort (Table 1). We did not observe a significant association of ETV4 with RFS of patients in the cohort.

**TABLE 1:** Summary table of the Cox proportional hazard model assessing the effect of ETV5 expression over adjCTX-treatment response in colon cancer patients.

			AdjCTX treated			AdjCTX untreated		
Dataset	Stage	Outcome variables	N	HR (C.I)	LRT (*p*-value)	N	HR (C.I)	LRT (*p*-value)
GSE39582	II/III	RFS	210	2.09 (1.29–3.40)	0.004019	262	1.38 (0.84–2.27)	0.21
GSE17536	III	DFS	56	6.05 (1.13–32.24)	0.03	—	—	—
GSE72970	IV	PFS	64	2.27 (1.18–4.4)	0.01	21	1.77 (0.21–1.48)	0.33
GSE39582	II/III	OS	210	2.22 (1.27–3.39)	0.007	267	1.11 (0.72–1.71)	0.6
GSE72970	IV	OS	63	2.14 (1.01–4.5)	0.05	21	1.78 (0.65–4.88)	0.30
GSE14333	II/III	DFS	61	2.27 (1.08–4.75)	0.03	86	1.21(0.47–1.46)	0.52

CI, confidence interval; DFS, disease-free survival; DSS, disease specific survival; HR, hazard ratio; LRT, log-likelihood ratio test (*p*-value); N, the numbers of cases; PFS, progression-free survival; RFS, relapse free-survival; The *p*-values have been calculated using cox-proportional hazard analysis.

Further, we validated the findings in colon cancer patients treated with adjuvant therapy in MCC, metastatic, and GSE14333 cohorts indicating that ETV5 is a strong predictor of disease-free survival. ETV5 expression also significantly associates with the overall survival of 5-FU treated patients in both CIT and metastatic cohorts (Table 1). We did not observe a significant effect of ETV5 over survival response of untreated patients (Table 1) suggesting that ETV5 does not predict a worse prognosis in colon cancer but predicts for poor adJCTX treatment response. We also observed a significant effect of ETV5 expression over the survival response of adjCTX-treated stage III patients (Supplementary Figure S1) suggesting that ETV5 is a useful response marker for therapy response on patients where it is widely used and impactful. Further, we also correlated ETV5 expression with clinically approved oncotype DX RS score to assess the clinical utility of ETV5 as a marker. ETV5 expression significantly correlates with oncotype DX recurrence score in all the datasets suggesting a clinical level prediction potential of ETV5 expression (Supplementary Table S2).

### ETV5 Expression Differs in Colon Cancer Patients With Proximal and Distal Tumors

**FIGURE 2:**
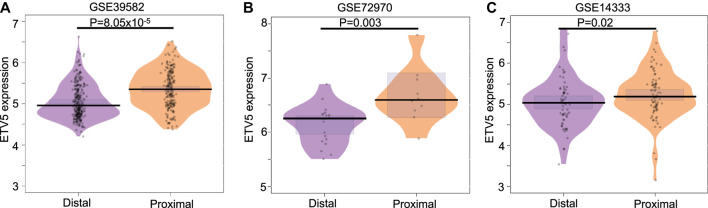
Comparison of ETV5 **(A–C)** expression in distal and proximal tumors of combined stage II and III colon cancer patients from CIT (GSE39582 left panel), metastatic (GSE72970 middle panel) and GSE14333 (right panel) cohorts. The *p*-value has been calculated using the Wilcoxon test.

After observing that ETV5 expression predicts the treatment response in colon cancer, we checked the expression level of ETV5 in stage II/III CC patients (both treated and untreated) with proximal and distal tumors as the gene expression can vary with the location of the tumor (Stintzing et al., 2017). We observed a significant difference in ETV5 expression in CIT program cohort (Wilcox test *p* = 8.05 × 10^–5^), metastasic cohort (GSE72970, Wilcox test *p* = 0.003) and GSE14333 cohort (Wilcox test *p* = 0.02, Figure 2). The result suggests a tumor-side specific role of ETV5 in colon cancer that can affect the underlying response to chemotherapy (Stintzing et al., 2017).

We also checked the correlation between ETV5 and colon cancer cell proliferating markers (MK167, MYC, MYBL2) in order to assess the effect of ETV5 over cell proliferation. We observed that ETV5 corelates with cell proliferation markers in adjCTX treated patients in most of the datasets (Supplementary Table S3) suggesting a significant role of ETV5 in cancer cell proliferation as observed in the colorectal cancer cells (Bazzocco et al., 2015) and other cancer cells (Puli et al., 2018; Mus et al., 2020).

## Discussion

5-FU-based adjCTX after surgery is the primary choice of treatment in early-stage colon cancer due to survival advantage over surgery alone (Wilkinson et al., 2010; Argiles et al., 2020). However, higher toxicity and inability to segregate responders from nonresponders using available markers is a notable challenge to the clinical success of therapy. Therefore, there is an urgent need to identify additional predictive markers for the 5FU-based-adjCTX response in colon cancer. To address the need, we investigated the expression regulation of members of the PEA3 subfamily of ETS transcription factors by DNA methylation and the potential of their expression as a predictive marker for chemotherapy response in stage II and III colon cancer patients using five publicly available independent colon cancer datasets.

Our analysis revealed higher expression of ETV4 and ETV5 genes in colon cancer tissue compared to normal tissue in TCGA samples (Figures 1A,B). Further, ETV4 expression showed a negative correlation with promoter methylation (Figure 1E) suggesting that DNA methylation mediates expression by hindering transcription factor binding (Esteller, 2007). Additionally, we observed a paradoxical positive correlation between ETV5 promoter hypermethylation (Figure 1F) and higher expression in cancer tissue (Figure 1B) suggesting that hypermethylation may facilitate gene expression either by the opening of chromatin (Smith et al., 2020) or mechanical inhibition of transcriptional repressor binding (Nabilsi et al., 2009) or allowing transcription from an alternative promoter (Renaud et al., 2007). To our knowledge, there has been no study exploring the methylation-expression relation of PEA3 member proteins in colon cancers, and the results need to be validated in independent colon cancer cohorts. Further, a detailed mechanistic study in higher experimental model systems is needed on how the promoter hypermethylation increases the gene expression.

Additionally, we explored the role of ETV4 and ETV5 in 5-FU-based-adjCTX response prediction utilizing three publicly available colon cancer patient cohorts. Survival analysis revealed that higher ETV5 expression significantly associated with shorter RFS/DFS/PFS in colon cancer in stage II and III patients (Table 1). ETV5 expression significantly but moderately correlated with oncotype DX recurrence score (Supplementary Table S2) suggesting that prediction based on ETV5 expression will be in line with the standard response prediction tools like oncotype Dx score. It further confirms that use of ETV5 along with other known predictors for adjCTX response can improve the prediction accuracy.

Further, we observed higher expression of ETV5 in the proximal tumor as compared to the distal tumor (Figures 2A,B) in all three (CIT, GSE14333 and GSE72970 cohorts) which is in accordance with more aggressive and high-grade histology of proximal tumors compared to distal tumors (Stintzing et al., 2017). We also observed significant correlation between ETV5 expression and cell proliferating marker gene expression (Supplementary Table S3) similar to the earlier observation as higher ETV5 expression has been associated with faster cell proliferation and aggressive phenotypes (Bazzocco et al., 2015). ETV5 overexpression stimulates CRC angiogenesis through activation of VGFR by PDGFR-β/Src/STAT3 signaling (Llaurado et al., 2012) and increases bevacizumab resistance through AKT, ERK, and p38 signaling decreasing overall survival of the patients (Llaurado et al., 2012). However, studies in larger human cohort and animal model systems can fully explain the detailed mechanism for the ETV5 role in 5-FU-based adjCTX resistance. Further, the association of ETV5 expression with drug response in patients with different clinical (e.g., number of nodes, location of metastasis) and molecular features (e.g., mutations types) also need to evaluated using appropriate cohorts.

The current study identified ETV5 as a biomarker of 5-FU-based adjCTX response in colon cancer patients with evidence II level as defined by Simon et al. (2009), and revealed that higher ETV5 is associated with poor response in patients. These results suggest that ETV5 could be useful for the identification of responders before administration 5-FU-based adjCTX when included along with other already established clinicopathological markers.

## Data Availability

The original contributions presented in the study are included in the article/Supplementary Material, further inquiries can be directed to the corresponding author.
