# Supplementary material for: Higher ETV5 Expression Associates With Poor 5-Florouracil-Based Adjuvant Therapy Response in Colon Cancer
Source: Front Pharmacol. 2021 Feb 9;11:620811. doi: 10.3389/fphar.2020.620811 (PMC7917823; doi:10.3389/fphar.2020.620811)
Supplement: Supplementary file 1 [file datasheet1.pdf]

Suppl. Table 1: Clinical characteristic of studied patients

	CIT cohort (GSE39582)		MCC cohort (GSE17536)	Metastatic cohort (GSE72970)		GSE1433	GSE1433
Traits	Treated (210)	Untreated (262)	Treated (56)	Treated (64)	Unreated (21)	Treated (61)	Untreated (86)
Sex (M/F)	116/94	113/149	30/26	26/37	10/11	31/30	42/44
Location (Distal/proximal)	137/73	142/120	-	43/20	9/12	26/35	40/46
Stage (II/III/IV)	58/152/0	205/57/0	0/56/0	0/0/64	0/0/21	13/48/0	63/23/0

Suppl. Table 2: Correlation analysis between ETV5 and oncotype DX recurrence score in the studied cohorts

Dataset	Correlation(r)	P
GSE39582	0.45	$6.28x10^{-12}$
GSE17536	0.52	$3.91x10^{-5}$
GSE72970	0.27	0.03
GSE14333	0.49	$7.44 x10^{-5}$

The Pearson correlation coefficients (r) and respective p values (p) have been shown in the table.

Suppl. Table 3: Correlation analysis between ETV5 and cell proliferation markers (MK167, MYC and MYBL2) in the adjCTX treated samples from studied cohorts

	Gene	MKI67	MYC	MYBL2
GSE14333	ETV5	-0.32 (0.01)	-0.32 (0.02)	-0.42 ($7.87 x10^{-4}$)
GSE72970	ETV5	-0.03 (0.79)	-0.33(0.01)	-0.25(0.05)
GSE39582	ETV5	0.06(0.40)	-0.18 ($9.3x10^{-3}$)	-0.08 (0.27)
GSE17536	ETV5	-0.27 (0.05)	-0.37($4.92x10^{-3)}$	-0.34 (0.01)

The Pearson correlation coefficients (r) and respective p values (p) have been shown in the table in the parenthesis.

Suppl. Figure 1: Effect of ETV5 expression over stage III colon cancer patients in different cohort

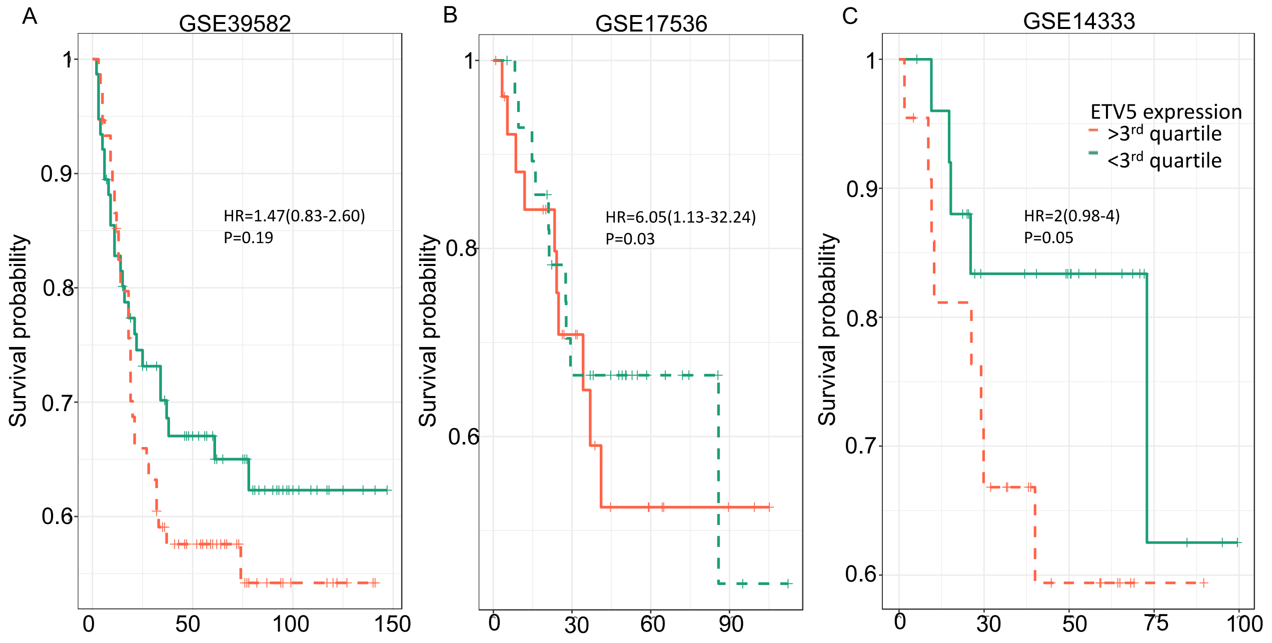

Effect of ETV5 expression over RFS in 5-FU-based adjCTXtreated stage III colon cancer patients. For visualization purpose, patients with the ETV5 expression > 3rd quartile has been categorized as high (red) and with expression <1st quartile has been categorized as low (low). However, the hazard ratio and p-value have been calculated using cox-regression for the all samples together (without categorization) and has been shown in the figure.
